# Optimizing Care in Primary Biliary Cholangitis: Current Treatments and the Second-Line Decision

**DOI:** 10.1007/s10620-025-09537-3

**Published:** 2025-11-21

**Authors:** Gina Choi, Arun B. Jesudian, Sammy Saab

**Affiliations:** 1 Departments of Medicine and Surgery, David Geffen School of Medicine at UCLA, Los Angeles, CA USA; 2 UCLA Medical Center, Pfleger Liver Institute, Los Angeles, CA USA; 3 Division of Gastroenterology and Hepatology, Weill Cornell Medicine, New York, NY USA

**Keywords:** Primary biliary cholangitis, Ursodeoxycholic acid, Elafibranor, Seladelpar, Alkaline phosphatase, Pruritus, Treatment guidelines

## Abstract

Primary biliary cholangitis (PBC) is a chronic, autoimmune, cholestatic liver disease characterized by small bile duct injury that can lead to advanced liver disease including cirrhosis and liver failure. Early diagnosis of PBC is crucial to mitigate the onset of advanced liver disease. However, even after diagnosis, some patients with PBC remain untreated or continue to progress to advanced disease despite initiation of treatment. Further, extrahepatic symptoms such as pruritus and fatigue substantially affect quality of life yet may not be addressed with standard treatment. Ursodeoxycholic acid (UDCA) stands as the first-line treatment for PBC and has been shown to increase survival and reduce the need for liver transplantation. Nonetheless, some patients have an inadequate response or are intolerant to UDCA. Furthermore, UDCA does not address pruritus or fatigue. Approved second-line therapies for PBC include elafibranor and seladelpar. Elafibranor and seladelpar were recently granted Food and Drug Administration (FDA) approval in the accelerated pathway after reducing alkaline phosphatase (ALP) levels and showing promise in improving patient-reported symptoms such as pruritus and fatigue, though they are not without adverse events. With the availability of these new, second-line therapies, current treatment guidelines should be updated to emphasize early evaluation of treatment response to UDCA and to incorporate patient-reported outcomes that impact symptom burden and quality of life. With the current emphasis on patient engagement and personalized medicine, clinicians should consider evaluating symptom burden at diagnosis and improving quality of life to be as important as ALP levels in terms of successful PBC treatment.

## Introduction

Primary biliary cholangitis (PBC) is a chronic, autoimmune, cholestatic liver disease characterized by injury to the small bile ducts [1, 2]. When left untreated, immune-mediated degeneration of the bile ducts impairs bile flow, leading to advanced liver diseases, such as cirrhosis and liver failure [2–4]. In addition to liver damage, up to 80% of patients living with PBC experience pruritus and fatigue, both of which significantly impact their quality of life [5–7]. PBC was previously called “primary biliary cirrhosis” until international liver societies agreed to “correct the inaccuracy” and “remove the cirrhosis stigma” to better serve and respect patients [8].

The precise etiology of PBC is not fully understood, but a combination of genetic predisposition and environmental exposures is implicated in its development [5, 9]. A variety of autoimmune-related gene clusters and PBC-specific deregulated genes contribute to disease susceptibility, and close family members of patients with PBC are at increased risk compared with distant relatives [9, 10]. Additionally, high exposure to industrial pollutants and smoking is associated with the risk of PBC [10, 11].

Globally, different studies have reported a wide range for the incidence and prevalence of PBC, with studies from North America and Europe showing a higher prevalence (21.81 per 100,000 persons and 14.59 per 100,000 persons, respectively) compared with studies conducted in the Asia–Pacific region (9.82 per 100,000 persons) [12, 13]. The incidence and prevalence of PBC is also higher in women than men, with a prevalence of 57.8 per 100,000 women compared with 15.4 per 100,000 men; thus, a diagnosis of PBC is predominantly made in middle-aged, White women [6, 14]. However, recent studies suggest that the prevalence and incidence of PBC are increasing across many populations, and both the prevalence and incidence are increasing faster in men than in women [6, 12, 14].

In PBC, certain populations face an increased risk of developing advanced liver disease [15]. For instance, Hispanic patients with PBC are more likely to develop abnormal liver biochemistries, have overlap syndrome with autoimmune hepatitis, and experience variceal bleeding or ascites when compared with non-Hispanic patients with PBC [15]. In addition, men with PBC have higher mortality rates than women, possibly due to later diagnosis, as men are more likely to be asymptomatic, or due to a lower clinical suspicion of PBC in men among providers [16]. Racial and ethnic disparities also exist, with the Hispanic population experiencing reduced access to PBC care and higher rates of comorbidities [15, 17, 18]. Comorbid conditions, such as osteoporosis, Sjögren’s syndrome, metabolic syndrome, and autoimmune diseases such as autoimmune hepatitis and perhaps celiac disease, are commonly observed in patients with PBC and further complicate disease management [19]. Metabolic dysfunction–associated steatotic liver disease occurs in about 30% of patients with PBC and is associated with worse treatment outcomes and prognosis [20].

The rising prevalence of PBC coincides with the advent of new treatment options and a renewed effort to find consensus on key areas of disease management [12–14]. These include updates to diagnostic criteria, the importance of quality-of-life outcomes, early evaluation of the response to first-line treatment with ursodeoxycholic acid (UDCA), and criteria for initiating the use of second-line therapies [21]. In this review, we discuss the evolving disease and treatment landscape and highlight the need to update current guidelines on the management of PBC.

## Diagnosis of PBC and the Benefits of Early Treatment

Early identification of PBC is increasingly common due to greater disease awareness among clinicians and the increased use of serum alkaline phosphatase (ALP) tests [11, 12]. A diagnosis of PBC is often made in patients between 40 and 60 years of age, typically following the discovery of elevated ALP levels [6, 11, 19]. Many patients are diagnosed when elevated ALP levels are found incidentally during routine monitoring by primary care physicians or specialists [1, 4, 22]. If PBC is suspected, a positive test for serum antimitochondrial antibody (AMA)—a hallmark of PBC—can be used to confirm the diagnosis [19, 23]. Some patients are AMA-positive but lack elevations in ALP or gamma-glutamyl transferase; these patients are at a relatively low risk for disease progression [24]. For patients who are AMA-negative, additional testing for PBC-specific antinuclear antibodies, such as anti-sp100 and anti-gp210, or anti-smooth muscle antibodies (ASMA), may also be useful in supporting a diagnosis of PBC [25, 26]. Of note, positive anti-gp210 has been associated with poor outcomes and further disease progression in PBC [27]. Patients who are AMA-negative often have elevated ANA and ASMA, lower serum IgM levels, less pruritus, and more nonhepatic autoimmune disorders [28, 29].

Liver stiffness measurements at PBC diagnosis are increasingly utilized, as progressive liver stiffness indicates increasing fibrosis and is associated with poor outcomes [19, 30]. Noninvasive methods, such as vibration-controlled transient elastography (VCTE), have increased the feasibility of routine liver stiffness testing at PBC diagnosis, with a score of ≥ 10 kPa indicating severe fibrosis [31]. Liver stiffness measurement by VCTE improves outcome prediction in PBC; patients with 8–15 kPa or higher at diagnosis should be treated more aggressively than those with < 8 kPa [30]. Invasive procedures like liver biopsy are no longer routine for initial diagnosis but may be useful in select cases, such as in patients who are seronegative or in those who are suspected of having overlapping syndromes or other conditions, including autoimmune hepatitis or drug-induced liver injury [19].

Although symptoms of pruritus and fatigue are associated with PBC, they are not diagnostic of the disease, and disease severity does not necessarily correlate with the severity of these symptoms [4, 31, 32]. Asymptomatic patients (though not as common as once believed) have better overall outcomes compared with those who exhibit symptoms; however, most patients with PBC will progress to developing symptoms within two to four years if left untreated, highlighting the need for early diagnosis and treatment [19]. Real-world evidence suggests that even after diagnosis, some patients with PBC continue to progress to advanced disease stages, with up to 1 in 7 people remaining untreated after diagnosis despite the availability of effective treatments [33, 34].

Notably, growing evidence suggests that people with PBC can be stratified at diagnosis based on risk factors and clinical characteristics that predict outcomes [31, 35]. For instance, male sex and younger age at diagnosis are associated with increased disease progression, and large-scale studies show that ALP levels at diagnosis are prognostic of liver transplantation and death [22, 36]. In addition, liver stiffness at diagnosis is predictive of disease progression, even in patients with PBC who receive UDCA, which is the recommended first-line treatment for nearly all patients with PBC, regardless of disease stage or other baseline factors [19, 30, 36, 37]. Thus, additional research is needed to understand PBC prognosis in the context of response to treatment.

## Current Treatment Algorithm in PBC

### Initial Treatment and Assessment of Treatment Response

Weight-based UDCA is the initial treatment for patients with PBC and has been shown to significantly increase survival and reduce the need for liver transplantation [19, 38, 39]. UDCA is a hydrophilic bile acid that reduces the accumulation of toxic bile acids, promotes bile flow, and reduces liver inflammation [19, 40]. However, some patients with PBC have an inadequate response to UDCA treatment, and others are intolerant to UDCA. In addition, treatment with UDCA does not improve extrahepatic symptoms such as pruritus or fatigue in patients with PBC [41, 42].

Historically, response to UDCA treatment has been assessed after 12 months of therapy [31, 43]. Over time, various response criteria have been developed to determine treatment efficacy. These criteria are based on liver biochemical values, primarily serum ALP and total bilirubin, and categorize patients with PBC either as responders or non-responders (binary criteria) or by predicted outcomes associated with their biochemical response (continuous criteria) [19]. Among the commonly recommended response criteria, two key metrics are widely used: 1) an ALP threshold of 1.67 × the upper limit of normal (ULN) and 2) the GLOBE score, which accounts for age along with laboratory values for total bilirubin, ALP, albumin, and platelet count [41]. Regardless of the criteria used, approximately 20% to 30%, and in some studies up to 40% of patients with PBC demonstrate an inadequate response to UDCA after one year of treatment [19, 39, 43, 44]. An additional 5% of patients with PBC are intolerant to UDCA, with the most common reasons for discontinuation being alopecia and diarrhea [45, 46].

In a study of the GLOBAL PBC Study Group (an international database of 17 liver centers across North America and Europe), the POISE criteria, which include ALP levels of < 1.67 × ULN and normal total bilirubin levels at one year, were used to evaluate the response to UDCA treatment at six months and one year [47]. ALP values were used as an indicator of insufficient response to UDCA treatment. The study found that an ALP cutoff of 1.9 × ULN could be used to identify non-responders to UDCA as early as six months into treatment rather than at one year [47]. Utilizing this updated metric would allow for earlier identification of patients with an inadequate response to UDCA treatment, particularly in those with advanced disease who may benefit from early initiation of second-line therapy [31, 35, 47].

In addition, while most patients with PBC treated with UDCA exhibit a decrease in ALP levels, those who achieve ALP normalization have higher transplant-free survival [48, 49]. However, only 1 in 5 patients with PBC treated with UDCA for one to two years achieve ALP normalization with no other liver abnormalities [19, 35]. As such, there remains a need for longitudinal studies that evaluate the real-world benefit of early transition from first-line treatment with UDCA to second-line therapy. Also, despite some patients achieving a biochemical response during treatment with UDCA, many patients with PBC continue to experience a high symptom burden, particularly pruritus and fatigue, underscoring the need for a more comprehensive treatment approach for these individuals [50].

### Second-Line Therapies and Off-Label Treatment

The treatment landscape of PBC is rapidly evolving with multiple second-line therapies being newly approved [51]. It is essential for healthcare providers and patients with PBC, who do not have successful disease management with UDCA, to understand these new treatment options and their use in different patient populations (Table 1).

**Table 1:** Approved second-line therapies for PBC in different patient populations [52–61]

Agent	OCA	Elafibranor	Seladelpar
MOA	FXR agonist	Dual alpha delta PPAR agonist	PPAR delta agonist
Pivotal phase 3 clinical trial	POISE trial (NCT01473524)	ELATIVE trial (NCT04526665)	RESPONSE trial (NCT04620733)
Current regulatory decisions	US FDA approved but recently voluntarily withdrawn from the US market, EMA authorization revoked in 2024, UK MHRA approved	US FDA approved, EMA approved, UK MHRA approved	US FDA approved, EMA approved, UK MHRA approved
Efficacy	Primary outcome measure: Composite biochemical response (ALP < 1.67 × ULN, ≥ 15% reduction in ALP from baseline, and total bilirubin ≤ ULN) at 12 months		
Percent of patients achieving the primary outcome	46% (5–10 mg dose) 47% (10 mg dose) 10% (placebo)	51% (80 mg dose) 4% (placebo)	62% (10 mg dose) 20% (placebo)
Important information for patients			
Warnings and precautions	OCA has been associated with hepatic decompensation and failure in patients with PBC with cirrhosis, severe pruritus, and reductions in HDL-C	Elafibranor has been associated with myalgia/myopathy, fractures, and fetal harm, but does not appear to induce or exacerbate pruritus	Seladelpar has been associated with fractures and should be avoided in patients with complete biliary obstruction or those receiving concomitant OAT3 or strong CYP2C9 inhibitors
Drug interactions	Warfarin CYP1A2 substrates Bile salt efflux pump inhibitors*	Hormonal contraceptives HMG-CoA reductase inhibitors Rifampin Bile acid sequestrants	OAT3 inhibitors* Strong CYP2C9 inhibitors* Rifampin CYP2C9 & CYP3A4 inhibitors BCRP inhibitors Bile acid sequestrants
Use in specific populations	Hepatic impairment—use is contraindicated	Lactation—recommend against breastfeeding during and for 3 weeks after the final dose Hepatic impairment—use is not recommended in patients with decompensated cirrhosis	Hepatic impairment—use of seladelpar is not recommended in patients with decompensated cirrhosis

*ALP* alkaline phosphatase, *BCRP* breast cancer resistance protein, *CYP* cytochrome P450, *EMA* European Medicines Agency, *FDA* Food and Drug Administration, *FXR* farnesoid X-activated receptor, *HDL-C* high-density lipoprotein cholesterol, *HMG-CoA* 3-hydroxy-3-methylglutaryl-coenzyme A, *MHRA* Medicines and Healthcare products Regulatory Agency, *MOA* mechanism of action, *OAT3* organic anion transporter 3, *OCA* obeticholic acid, *PBC* primary biliary cholangitis, *PPAR* peroxisome proliferator-activated receptor, *ULN* upper limit of normal

*Denotes drug interaction with a recommendation to avoid concomitant use; remaining drug interactions have a recommendation to monitor with concomitant use

#### Obeticholic Acid

Obeticholic acid (OCA) is a farnesoid X-activated receptor agonist, which reduces the buildup of toxic bile acids [62]. OCA was first approved by the US Food and Drug Administration (FDA) in 2016 as a second-line combination therapy for patients with PBC with an inadequate response to UDCA, or as monotherapy for patients who are intolerant to UDCA [19]. However, it was recently voluntarily withdrawn from the US market following a request from the FDA [63]. Its authorization was also revoked by the European Medicines Agency (EMA) in 2024, but it is still approved by the UK Medicines and Healthcare products Regulatory Agency (MHRA) [64, 65].

In the placebo-controlled, phase 3 POISE trial (NCT01473524), 46% of patients with PBC who were treated with OCA 5–10 mg and 47% of patients treated with OCA 10 mg met the primary endpoint of a composite biochemical response (ALP < 1.67 × ULN, ≥ 15% reduction in ALP from baseline, and total bilirubin ≤ ULN) compared with 10% in the placebo group [44, 52]. However, increased pruritus was a common adverse event associated with OCA treatment [19]. Additionally, dose-dependent reductions in high-density lipoprotein cholesterol were observed with OCA [53]. Other treatment-emergent adverse events following OCA 10 mg treatment in clinical trials included fatigue (23%), nasopharyngitis (18%), and nausea (11%) [52]. Twelve percent of patients receiving OCA 10 mg discontinued from the study compared with 4% in the placebo arm [53]. OCA is also contraindicated in patients with decompensated cirrhosis, compensated cirrhosis with portal hypertension, or complete biliary obstruction [53], and frequent monitoring of liver function while on treatment is recommended [53, 66–68].

#### Elafibranor

Elafibranor is a dual alpha and delta peroxisome proliferator-activated receptor (PPAR) agonist approved by the US FDA (under the accelerated pathway), the EMA, and the UK MHRA as a second-line combination therapy for patients with PBC with an inadequate response to UDCA, or as monotherapy for patients who are intolerant to UDCA [54, 55, 69]. In the placebo-controlled, phase 3 ELATIVE trial (NCT04526665), 51% of patients treated with elafibranor 80 mg achieved the primary endpoint of a composite biochemical response (ALP < 1.67 × ULN, ≥ 15% reduction in ALP from baseline, and total bilirubin ≤ ULN) compared with 4% of patients who received placebo [56]. In the same trial, patients with PBC and moderate to severe pruritus receiving elafibranor did not show a significant difference from those receiving placebo in the mean change from baseline in the Worst Itch Numeric Rating Scale (NRS) score [56]. However, patients with PBC in the ELATIVE open-label extension analysis showed clinically meaningful improvements in both sleep and fatigue, as measured by the Epworth Sleepiness Scale and both the Patient-Reported Outcomes Measurement Information System Fatigue Short Form 7a and PBC-40 Fatigue domain, respectively [70]. Regarding safety, in the ELATIVE trial, elevated creatine phosphokinase levels and muscle injury were more common among patients receiving elafibranor when compared with those receiving placebo [56]. Other treatment-emergent adverse events following elafibranor treatment in the ELATIVE trial included COVID-19 (29%), pruritus (20%), and abnormal weight gain (19%) [56]. Further, 10% of patients receiving elafibranor in the ELATIVE trial discontinued due to adverse events compared with 9% in the placebo arm [56].

#### Seladelpar

Seladelpar is a selective PPAR delta agonist [56, 70] approved by the US FDA (under the accelerated pathway), the EMA, and the UK MHRA as second-line combination therapy for patients with PBC with an inadequate response to UDCA, or as monotherapy for patients who are intolerant to UDCA [57–60]. In the placebo-controlled, phase 3 RESPONSE trial (NCT04620733), 62% of patients treated with seladelpar 10 mg achieved a composite biochemical response (ALP < 1.67 × ULN, ≥ 15% reduction in ALP from baseline, and total bilirubin ≤ ULN) compared with 20% in the placebo group [60]. In the same trial, patients with PBC and moderate to severe pruritus receiving seladelpar showed a significant reduction from baseline in the pruritus NRS score (-3.2 points) when compared with placebo (-1.7 points) [60]. Seladelpar has also led to significant decreases in fatigue and sleep disturbance, as measured by the PBC-40, in patients with severe pruritus [71]. Regarding safety, in the RESPONSE trial, COVID-19 infection, headache, abdominal pain, nausea, and abdominal distention were more common in the seladelpar group than in the placebo group [60]. In the RESPONSE trial, 3% of patients in the seladelpar group discontinued the study drug due to adverse events compared with 5% in the placebo group [60].

#### Fibrates

Fibrates are sometimes used as an off-label alternative for patients with PBC who have an inadequate response to UDCA [68]. Fenofibrate, which is a lipid-lowering medication, is a broad PPAR activator that has anti-inflammatory and antifibrotic properties. However, its use is discouraged in patients with decompensated cirrhosis due to potential worsening of disease [19, 31, 68]. Additionally, long-term fibrate use is associated with renal dysfunction, which may require discontinuation [51, 72].

Bezafibrate is a type of fibrate that is sometimes used in Canada, Europe, South America, and Asia in combination with UDCA. Safety concerns, such as rhabdomyolysis (rare), myalgias, serum creatinine elevations (reversible with dose reduction), and aminotransferase elevations, limit its use [19, 73]. In a clinical trial of bezafibrate, temporary or permanent discontinuation of the drug occurred in 7 patients (14%) [73].

Real-world studies evaluating the combination of UDCA, OCA, and fibrates indicate that some patients with PBC may benefit from this approach, although data from randomized controlled trials are needed to confirm the efficacy of combining these therapies [74]. Robust, phase 3 data are needed to further consider fibrates as a viable second-line therapy option.

### Summary of Current Treatments in PBC

**Fig. 1:**
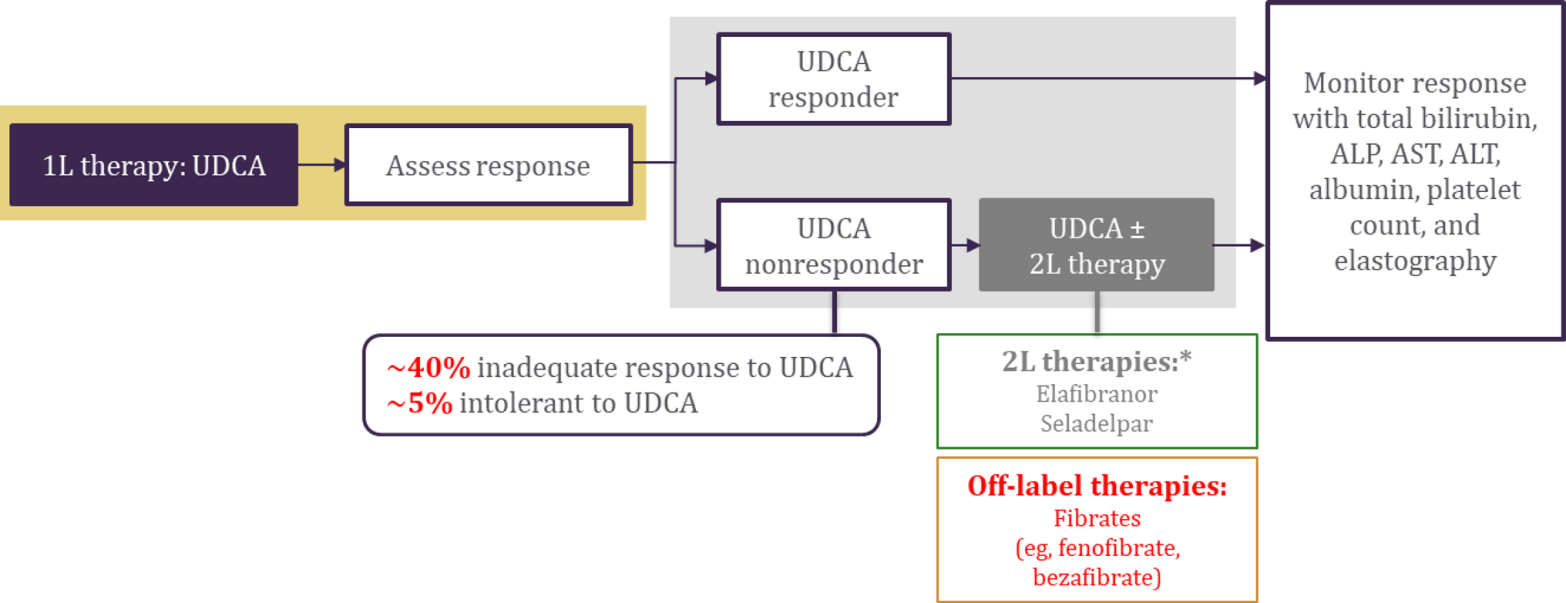
Treatment algorithm for patients with primary biliary cholangitis. *Obeticholic acid was voluntarily withdrawn from the US market, and EMA authorization was revoked in 2024, but it is still approved by the UK MHRA [63–65]. *1L* first-line, *2L* second-line, *ALP* alkaline phosphatase, *ALT* alanine aminotransferase, *AST* aspartate aminotransferase, *EMA* European Medicines Agency, *MHRA* Medicines and Healthcare products Regulatory Agency, *UDCA* ursodeoxycholic acid

As shown in Fig. 1, the current treatment algorithm for patients with PBC includes initiating first-line UDCA (at a dose of 13–15 mg/kg/day) and assessing the treatment response, as up to 40% of patients have an inadequate response to UDCA treatment, and 5% of patients are intolerant to UDCA [19, 45]. Response to UDCA treatment should be continually assessed up to 12 months, with recent evidence showing that UDCA responders and those with an inadequate response to UDCA can be distinguished as early as six months into treatment [19, 47]. For patients with PBC who have an inadequate response to UDCA, early initiation of UDCA in combination with second-line therapy, such as elafibranor or seladelpar, should be considered [19, 57, 61]. These second-line therapies may alternatively be given as monotherapy for patients who are intolerant to UDCA. In both UDCA responders and those who initiate second-line therapy, monitoring of the treatment response should continue with frequent and consistent assessment of total bilirubin, ALP, aspartate aminotransferase, alanine aminotransferase, albumin, platelet count, and elastography measurements [19].

## Evolving Treatment Goals and Outcomes in PBC

With the availability of new, second-line therapies, current treatment guidelines (Table 2) should be updated to emphasize early evaluation of the treatment response to UDCA and the identification of patients with PBC who have an inadequate response; these updates should also define the criteria to be used for the initiation of second-line therapy [19, 68]. Although patients with PBC who are treated with UDCA show a reduced risk of liver transplantation, studies indicate that approximately 25% of patients continue to experience PBC-related pruritus, while approximately 34% of patients report PBC-related fatigue [78]. This emphasizes the lack of addressing patient-centric symptoms like pruritus and fatigue when only UDCA is considered as a treatment option. Updated treatment algorithms should incorporate patient-reported outcomes, such as symptom burden and quality of life, into clinical treatment guidelines [21, 79, 80]. Management of Sjögren’s syndrome symptoms, bone mineral loss, pruritus, and fatigue can help address quality of life concerns in patients with PBC [5, 41]. Current emphasis on patient engagement and personalized medicine may mean that considering symptom burden at diagnosis and improving patient quality of life are meaningful and as important as ALP levels in terms of successful PBC treatment.

**Table 2:** Summary of current treatment guidelines in PBC

Governing body	AASLD	ACG-CLDF	APASL	CSH	EASL
Title	Primary Biliary Cholangitis: 2018 Practice Guidance from the American Association For the Study of Liver Diseases	Diagnosis and Management of Primary Biliary Cholangitis	APASL Clinical Practice Guidance: The Diagnosis and Management of Patients With Primary Biliary Cholangitis	Guidelines on the Diagnosis and Management of Primary Biliary Cholangitis (2021)	EASL Clinical Practice Guidelines: The Diagnosis and Management of Patients With Primary Biliary Cholangitis
Year published	2019 [19]	2019 [4]	2022 [75]	2023 [76]	2017 [77]
Key recommendations	Comprehensive review of the natural history of PBC, diagnosis, treatment guidelines, and overall considerations	Provided an expert opinion on initial diagnosis, treatment options and prognosis, and symptom management	Comprehensive review of PBC disease landscape and pathogenesis, and review of treatment response criteria	Provided an expert panel opinion on all aspects of PBC, including preclinical PBC treatment and sex-specific considerations	Review of diagnosis and treatment, with specific attention to treatment algorithms and screening
Year updated	2022 [68]	2023 [31]	N/A	N/A	N/A
Key updates	Updated guidance on off-label fibrate use, and caution on OCA use in patients with advanced cirrhosis	Provided updated guidance on liver staging at diagnosis and a treatment algorithm, which included recent second-line treatment options			
Years since last update	3	2	3	2	8

*AASLD* American Association for the Study of Liver Diseases, *ACG-CLDF* American College of Gastroenterology–Chronic Liver Disease Foundation, *APASL* Asian-Pacific Association for the Study of the Liver, *CSH* Chinese Society of Hepatology, *EASL* European Association for the Study of the Liver, *N/A* not applicable, *OCA* obeticholic acid, *PBC* primary biliary cholangitis

Traditionally, the primary goal of PBC treatment has been to arrest disease progression and stabilize disease by reducing ALP levels; however, ALP normalization, and not simply ALP reduction, is emerging as an optimal treatment goal for patients with PBC [49, 74]. In real-world studies, patients treated with long-term (at least 12 months) UDCA had better complication-free survival with ALP ≤ 1 × ULN compared with those in the 1–1.5 × ULN range; this benefit was even greater in high-risk patients, such as younger patients with PBC and those with more significant liver stiffness [49, 81]. Additionally, total bilirubin reduction to ≤ 0.6 × ULN has been associated with a lower risk of transplantation/death [82]. Future clinical trials connecting these criteria to an optimal treatment response may more precisely identify those who have the most to benefit from second-line therapy [79]. In addition, prognostic tools, such as the GLOBE score, may be useful in identifying patients with PBC who would benefit from second-line therapy, although long-term, real-world data are needed to refine treatment strategies and ensure timely initiation of a second-line option [21, 44, 81, 83]. Lastly, fibrosis reversal remains an optimistic goal of PBC treatment, but achieving this goal has not been demonstrated with current treatment options in controlled phase 3 trials [52, 56, 60].

## Conclusions

The landscape of PBC management is shifting with new second-line treatment options providing effective alternatives for patients with an inadequate response to UDCA. Comprehensive assessments at PBC diagnosis that integrate liver biochemistry, symptom burden, and quality-of-life measurements, along with noninvasive liver evaluations, are needed to ensure correct staging of the disease and aid in the identification of potential treatment challenges, such as compliance issues due to adverse symptoms. After diagnosis, the treatment response to UDCA should be evaluated as early as six months, which would allow for prompt identification of patients with PBC who may benefit from the initiation of second-line therapy. Overall, treatment algorithms should also be updated to provide guidance on which criteria, such as ALP normalization and symptom relief, constitute an optimal response. In this rapidly evolving treatment landscape, adopting an individualized approach to treatment will become a key factor of care and ensure that patients with PBC who require second-line therapy start as early as possible.

## Data Availability

No datasets were generated or analyzed during the current study.
